# Developmental gene regulatory networks in sea urchins and what we can learn from them

**DOI:** 10.12688/f1000research.7381.1

**Published:** 2016-02-22

**Authors:** Megan L. Martik, Deirdre C. Lyons, David R. McClay

**Affiliations:** 1 Biology Department, Duke University, Durham, North Carolina, 27708, USA

**Keywords:** developmental gene regulatory networks, sea urchin, dgrn, patterning, morphogenetic cassetes

## Abstract

Sea urchin embryos begin zygotic transcription shortly after the egg is fertilized.  Throughout the cleavage stages a series of transcription factors are activated and, along with signaling through a number of pathways, at least 15 different cell types are specified by the beginning of gastrulation.  Experimentally, perturbation of contributing transcription factors, signals and receptors and their molecular consequences enabled the assembly of an extensive gene regulatory network model.  That effort, pioneered and led by Eric Davidson and his laboratory, with many additional insights provided by other laboratories, provided the sea urchin community with a valuable resource.  Here we describe the approaches used to enable the assembly of an advanced gene regulatory network model describing molecular diversification during early development.  We then provide examples to show how a relatively advanced authenticated network can be used as a tool for discovery of how diverse developmental mechanisms are controlled and work.

## The sea urchin developmental gene regulatory networks

Developmental gene regulatory networks (dGRNs) describe the sequential regulatory changes that specify and diversify the cells of an embryo. The genes included in dGRNs encode transcription factors, components of signal transduction pathways, and often effector genes as markers of differentiated cell states. Models of dGRNs are assembled on the basis of experimental perturbations of an embryo’s developmental program and are valuable for explaining how spatial and temporal information is encoded in a multicellular organism’s genome. dGRNs have the potential of providing a causal understanding of how upstream specification controls downstream events (i.e. differentiation or cell biological functions). As such, a dGRN can serve as a tool for developmental and cell biologists alike. This article describes how dGRNs are assembled using the sea urchin embryo as a model and includes recent insights into sea urchin development that have benefitted from the assembly exercise.

The early specification events of the sea urchin embryo have been extensively documented, resulting in increasingly well-understood dGRNs for each cell type. Sea urchin development is relatively simple, easy to observe, and experimentally tractable, and experimental outcomes are rapidly obtained. Prior to genome sequencing, a provisional sea urchin endomesodermal dGRN was assembled starting with a small number of transcription factors and signaling inputs ^1^. In 2006, the sea urchin genome was sequenced ^2^, and since that time, labs across the globe have added to and reinforced an understanding of the regulatory linkages in the dGRNs through gastrulation ^3– 9^. In contrast to typical “hairball” or “interactome” networks, where hypothetical interactions are based on statistical inference, each linkage of the sea urchin dGRN is based on several experimental approaches, spatial and temporal validation, and in many cases cis-regulatory analyses to confirm a direct regulatory connection. This effort has provided many valuable insights into network function and has led to novel explorations into mechanisms of sea urchin development. We begin by summarizing experiments that demonstrated how the sea urchin dGRN was, and continues to be, assembled. Then we briefly review how dGRNs have been used as tools to understand how embryonic patterning works, how morphogenesis is controlled, and how evolutionary processes have modified dGRNs.

## Developmental gene regulatory network assembly process

GRNs reflect the relationship between genes in a system. In a graphic depiction of GRNs, the expression of a transcription factor or a component of a signal transduction pathway is represented as a node. The edges or connections between nodes reflect the regulatory relationship between nodes over time. In GRN models, an upstream regulator is drawn with an output that either activates or represses expression of a downstream gene. At any given time after development begins, many transcription factors are expressed and are under regulatory control by transcription factors upstream of them. Over time, this produces a regulatory network with many properties. At any given time, a cell in an embryo is controlled by the unique regulatory “state” of its GRN. All identical cells will tend to be regulated by the same GRN state, and as cells diverge from one another, their regulatory states change. Signaling molecules produced by one cell pass to adjacent or nearby cells where their inputs alter GRN states in recipient cells. Within a GRN state, it is possible to tease apart relationships of transcription factors and identify sub-circuits designed to accomplish the tasks of development. Among the tasks uncovered in the sea urchin dGRN are a number of properties that are commonly seen when network systems are studied in detail ^10– 12^. For example, a “double-negative gate” (repression of a repressor) was identified to initiate specification of a specific cell type ^4, 13^. “Spatial exclusion” sub-circuits were found in cells at the time these cells diverged from one another toward differentiation into distinct cell types; in each of the two nascent cell types, there was a sub-circuit to exclude the other ^14^. “Community effect” signaling was identified such that Nodal reinforced Nodal signaling in neighboring ectodermal cells to maintain Nodal signal production in that community of ectodermal cells ^11, 15^. “Feed-back sub-circuits”, in which a downstream transcription factor feeds back to maintain activation of an upstream transcription factor, and “feed-forward sub-circuits”, in which an upstream transcription factor feeds forward to activate multiple sequential downstream steps, were found. Feed-back and feed-forward sub-circuits tended to stabilize regulatory states ^4^ and contribute to the unidirectional trajectory of development. Sub-circuits just upstream of differentiation were found that drive the system forward and contribute to the activation of genes necessary for differentiation and cell biological function (e.g., 6, 16– 18). These and other sub-circuits provide dGRNs with modular design features that control explicit functions. As details of dGRN topology were identified, they were shown to contribute impressively to a growing understanding of developmental mechanisms in many systems and are central to research in sea urchin development as a consequence.

**Figure 1.**
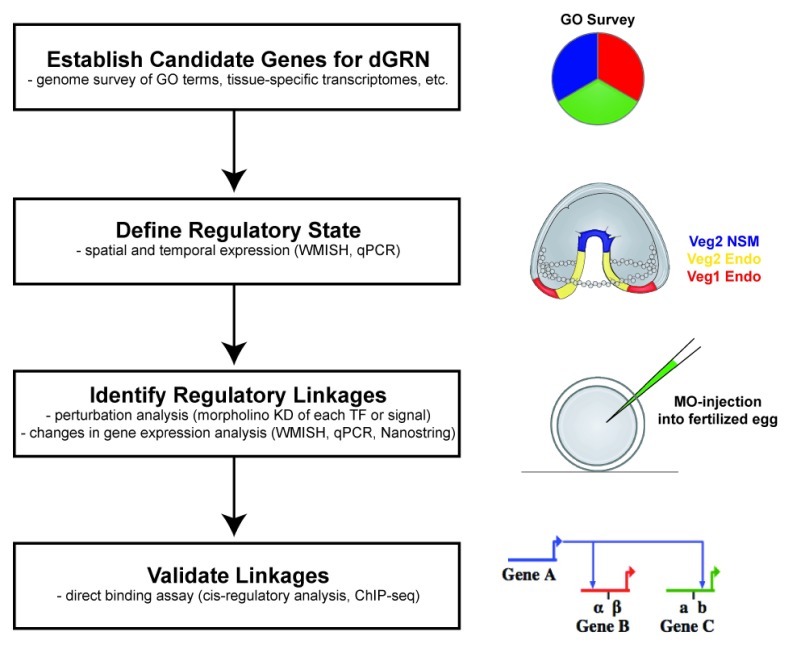
Steps in construction and validation of developmental gene regulatory networks (dGRNs). The process begins with identification of candidate molecules. Candidates for the sea urchin dGRN were defined as transcription factors or signal transduction pathway members that were expressed in spatiotemporally specific patterns in the embryo. The regulatory linkages were established by conducting perturbation analyses in which one candidate was perturbed and asking how its loss affected expression of other candidates. These established a preliminary dGRN model. That model was challenged in many ways, including testing predictions that gene A activated gene B and gene C through cis-regulatory analysis.

When an early draft of the *Strongylocentrotus purpuratus* genome became available, it provided an opportunity to identify hundreds of transcription factor genes and molecules of signal transduction pathways ( Figure 1). Each candidate development regulatory gene (transcription factors and signal transduction pathway gene) was assayed by quantitative polymerase chain reaction (qPCR) and whole-mount *in situ* hybridization (WMISH) to establish spatial and temporal expression patterns throughout early development ^19– 25^. Of the large number of transcription factors identified, those that were spatially or temporally distinct (or both) in expression in the early embryo were chosen for detailed efforts to define the distinct regulatory states of the embryo at a number of time points, beginning with fertilization and continuing through gastrulation. Ubiquitously expressed genes initially were excluded from the study with the notion that they were less likely to be involved in developmental regulatory decisions. Perturbation analyses of each identified signaling molecule and transcription factor then established linkages between nodes. Morpholino antisense oligonucleotides knocked down one transcription factor or signal, and analyses by qPCR, WMISH, or nanostring (or a combination of these) assayed the effect on other genes expressed at the same time, or in the same cell type, or both. This established a hierarchical relationship between the transcription factors, signals, and the genes encoding them in each cell type over time. The regulatory interactions were assembled into a network model by using BioTapestry as a platform for visualizing network topologies ^26, 27^. Many of the interactions were validated by using cis-regulatory analysis to determine direct binding of the transcription factor to specific sequences in the regulatory DNA of downstream genes. With this logical approach, hundreds of experiments were performed to establish sea urchin dGRN states in each emerging cell type up to gastrulation, and current efforts continue to extend the analysis to later time periods in development (e.g., 18, 28– 30).

## Patterning

**Figure 2.**
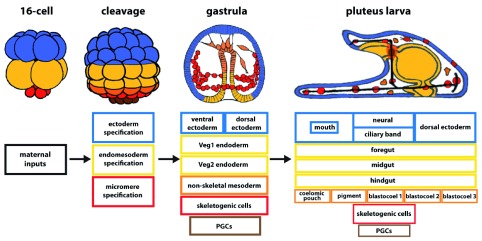
The patterning sequence of development results in cell diversification. Over the 24 hours from fertilization to the pluteus larval stage of *Lytechinus variagatus*, the number of developmental gene regulatory network (dGRN) states increases until there are more than 15 cell types in the early larva.

The term “patterning” describes processes that establish the body plan of an organism. Patterning processes provide “positional information” enabling cells to know their location in an embryo. These processes direct morphogenesis, and they provide short-range and long-range signaling inputs to accomplish construction of a three-dimensional plant or animal ( Figure 2). Each of these processes is driven by dGRNs. The sea urchin embryo begins patterning almost immediately after fertilization. By the 16-cell stage, zygotic transcription plus maternal inputs defines at least three distinct dGRN states ( Figure 2). At this time, the future mesoderm and endoderm are combined as an endomesoderm network state. A Delta-Notch signal separates progeny of these cells into mesoderm (cells that receive the Delta signal) and endoderm (endomesoderm progeny that do not receive the Delta signal) ^31– 33^, and further specification plus signaling subdivides the initial endomesoderm GRN state and ectoderm GRN state until there are at least 15 different cell types recognizable by early gastrulation ( Figure 2) ^7, 34^.

Signaling is integral for the patterning mechanisms that organize multicellular cell and tissue types. As specification progresses, signaling establishes the three axes of the embryo. Early patterning signals divide the embryo into regions along the animal-vegetal axis, established by asymmetrically localized maternal information, followed by a series of Wnt signaling events that progressively diversify cell identities along the anterior-posterior axis ^35– 41^. The anterior-posterior axis of the larva is approximately identical to the animal-vegetal axis of the egg. At about mid-cleavage, the dorsal-ventral (D-V) axis is specified through Nodal and bone morphogenetic protein (BMP) signaling ^15, 42, 43^, and toward the end of gastrulation, the right-left axis is established, again using Nodal and BMP and adding Hedgehog as a contributing signal ^16, 44– 47^. Thus, by the end of gastrulation, signaling inputs plus a progression of transcription factor activations establish dGRN states for each cell type in the embryo, and even within the same germ layer, patterning inputs provide localized cell identities.

Patterning continues to play a key role in organizing structures in the larva. As an example, a biomineralized skeleton provides the shape to the pluteus larva. To pattern that skeleton, signals from the ectoderm are received by the mesodermal skeletogenic cells, enabling them to position themselves correctly and to synthesize the calcium carbonate biomineral in the correct pattern ^48– 51^. Ectodermal signals are supplied from specific locations requiring the ectoderm to be patterned in advance. Ectodermal positional information leads to specification of two lateral patches of ectodermal cells specialized in secretion of vascular endothelial growth factor (VEGF) and fibroblast growth factor (FGF) ^5, 6, 8, 49, 50^. To provide that positional information, orthogonal bands of ectoderm are specified by even earlier signals. One of the ectodermal bands, the ciliary band, is specified at the boundary between the dorsal and ventral ectoderm as a consequence of Nodal (ventral) and BMP (dorsal) signaling ^15, 42, 52, 53^. BMP is synthesized in the ventral ectoderm and is transported, probably using Chordin as a chaperone, to induce dorsal ectoderm specification ^29, 53, 54^. The ciliary band then forms between the dorsal and ventral ectodermal territories and is further subdivided ^29^. The other ectodermal band, referred to as the border ectoderm, is specified in posterior ectoderm, just above the endoderm and orthogonal to the ciliary band separating the D-V ectoderm compartments. Wnt5 signaling from the endoderm is reported to induce the border ectoderm band in *Lytechinus* ^55, 56^, although a different Wnt is thought to be responsible in *S. purpuratus* ^56^, and that band also receives input from the Nodal-BMP signaling as seen in both *Lytechinus* and *Paracentrotus* ^30, 55^. The site where the ciliary band and the border ectoderm band intersect becomes the signaling center that produces VEGF and FGF, both of which are necessary for initiation and growth of the skeleton ^49– 51^. Other signals also provide patterning inputs into skeletogenesis ^58^. These data demonstrate that, in an organism, cells can be specified independently for long periods of time to seemingly establish independent dGRNs but that, at a later time, through signaling their dGRNs functionally intertwine once again. A good example is skeletal patterning, where signals from specific locations in the ectoderm provide patterning information and growth factors that direct the behavior of the mesoderm.

## Morphogenetic sub-circuits

**Figure 3.**
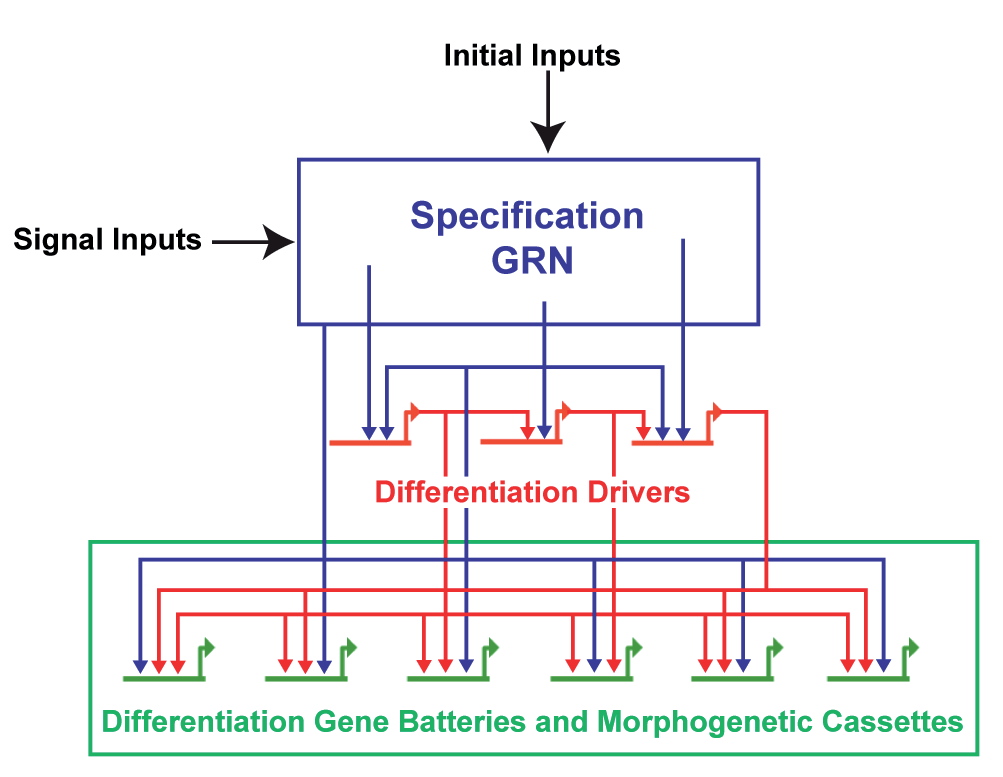
Process diagram of early development. Maternal inputs initiate specification. As cells divide, signaling becomes increasingly used to shape the specification sequence of each cell type. Toward the end of that process and proximal to differentiation, transcription factor sub-circuits drive expression of genes involved in differentiation and in morphogenesis.

Morphogenetic control circuitry is another area where the dGRN is valuable ( Figure 3). Each morphogenetic change incorporates multiple cell biological functions: changes in adhesion, motility, directionality, polarity, and so on. Perturbations that assess which transcription factors control individual cell’s biological properties have been valuable in dissecting details of those processes ^17, 18, 51, 56, 59– 62^. In an epithelial-mesenchymal transition (EMT), for example, five different dGRN sub-circuits control de-adhesion, motility, cell polarity, cell shape change, and invasion components of the EMT process ^61^.

Later in development, a specific feed-forward sub-circuit controls the directed migration of the presumptive primordial germ cells ^18^. Identification of such sub-circuits can then be used to penetrate the cell biology of each component process for each of the cell types (or organs) that have been defined in the larva. Along these lines, the circuits controlling ciliary band specification were identified, allowing for further analysis of the substrate for neural differentiation ^29^. Yet, other circuits that control myogenesis of the larva were recently identified ^28^. In each of these cases, the dGRN was valuable in gaining access to differentiation genes and effector genes ultimately controlled by that developmental GRN ( Figure 3).

## Evolution

A third valuable reason for studying dGRNs is to learn how evolutionary processes have changed dGRNs during species diversification. The sea urchin dGRNs are modeled primarily from data generated from three species of euechinoid sea urchins that are separated from one another by about 50 million years or less ^63^. Examination of more distantly related species reveals how rewiring of dGRNs correlates with evolutionary changes in morphology and cell behavior. For example, an examination of a cidaroid sea urchin, separated from the euechinoids by more than 255 million years, revealed significant rewiring of the dGRN that specifies the larval skeletogenic lineage ^64, 65^. In euechinoids such as *S. purpuratus* or *Lytechinus variagatus*, the skeletogenic lineage is specified early during cleavage stages, and after a species-specific number of cell divisions, all of the skeletogenic cells undergo EMT before gastrulation of the other mesodermal and endodermal lineages. In the cidaroid, *Eucidaris*, the number of skeletogenic cells varies from embryo to embryo, and the EMT of these skeletogenic cells does not occur until much later in development relative to *Lytechinus* ^66^. In *Strongylocentrotus* and *Lytechinus*, the skeletogenic lineage is specified by a core set of genes, including *Alx1*, *Tbr*, and *Ets1*, which are transcriptionally activated specifically in this lineage by unlocking a double-negative derepression sub-circuit controlled by *Pmar1* and *HesC* ^4^. In the cidaroid, *Eucidaris*, this double-negative derepression sub-circuit appears to be completely missing, and the spatiotemporal expression patterns of *Alx1*, *Tbr*, and *Ets1* are different as well ^64^. Thus, extensive rewiring of the dGRN occurs in sketogenic cell lineage, and morphogenesis of the skeletogenic cells differs, yet both modern sea urchins and the pencil urchins produce similar larval skeletons.

Comparative dGRN analysis can also reveal what aspects of specification are highly conserved during evolution. For example, experiments perturbing the function of *Alx1* in euechinoids, cideroids, and sea cucumber have shown that *Alx1* has a conserved role in promoting larval skeletogenesis ^64, 67^. At even larger evolutionary distance within echinoderms, valuable insights have been obtained through comparisons of skeletal, gut, and neural specification in sea stars compared with sea urchins. In each case, aspects of central dGRN circuitry were very similar despite about 500 million years of separation from the common ancestor ^68– 70^. dGRNs can also be used as a tool for understanding how circuitry has been co-opted during evolution. In a recent study, for example, it was learned that a conserved feed-forward sub-circuit involving Pax6, Six3, Six1/2, Eya, and Dach1 controls expression of the signal necessary for homing of primordial germ cells to the future gonad ^18^. That circuit is very similar to the feed-forward circuit that controls specification of the *Drosophila* eye and vertebrate muscle, suggesting that such circuits may in some cases evolve as units of function, in this case by providing a feed-forward device for directed cell migration.

## Conclusions

Sea urchin dGRNs describe the sequence of specification of all cells in the embryo up to the end of gastrulation. dGRN topology models produced in BioTapestry ( http://sugp.caltech.edu/endomes/) record the current status of the network in *S. purpuratus*. Much more than a graphic description, it reflects a large number of experiments, where each connection is supported by multiple tests of the hypothesis that the expression of gene A activates or represses gene B. In its current form, the sea urchin dGRN includes more than 100 transcription factors and a number of signaling pathways, and in most cases multiple laboratories have validated each connection in *S. purpuratus*, and most are the same in *Lytechinus* and *Paracentrotus*.

With a high level of confidence in the structure of the sea urchin dGRNs, they became useful as a tool for exploring many other developmental questions. Here, we describe how the dGRNs have been used to inform patterning mechanisms, especially those necessary to produce the larval skeleton. We show how they have been useful in gaining a greater understanding of an EMT and a directed cell movement mechanism, both components of morphogenesis. We also describe how dGRNs are used as tools for discovery of evolutionary relationships.

The growth in our understanding of dGRNs has provided ways to address many unanswered questions. The ability to trace the entire specification trajectory of a cell type until it terminally differentiates is now a realistic goal. That ability has enormous power because it allows one to interrogate, dissect, and understand how that cell type arises and how it works in detail. This will be valuable in understanding the entire history of neurogenesis, for example, and in determining the mechanisms by which neurons diversify toward different neurotransmitter cell types. Other cell types can be followed with the same goal. dGRNs will also help us to understand how the upstream circuitry controls other morphogenetic movements in the early embryo. Thus, the information in the dGRN is useful both for gaining an intrinsic understanding of how developmental control circuitry works and as a tool for understanding patterning, morphogenesis, and evolutionary change.

## References

[1] Davidson EH Rast JP Oliveri P: A genomic regulatory network for development. *Science.* 2002;295(5560):1669–78. 10.1126/science.1069883 11872831

[2] Sea Urchin Genome Sequencing Consortium, Sodergren E Weinstock GM: The genome of the sea urchin *Strongylocentrotus purpuratus*. *Science.* 2006;314(5801):941–52. 10.1126/science.1133609 17095691 PMC3159423

[3] Davidson EH Erwin DH: Gene regulatory networks and the evolution of animal body plans. *Science.* 2006;311(5762):796–800. 10.1126/science.1113832 16469913

[4] Oliveri P Tu Q Davidson EH: Global regulatory logic for specification of an embryonic cell lineage. *Proc Natl Acad Sci U S A.* 2008;105(16):5955–62. 10.1073/pnas.0711220105 18413610 PMC2329687

[5] Su YH Li E Geiss GK: A perturbation model of the gene regulatory network for oral and aboral ectoderm specification in the sea urchin embryo. *Dev Biol.* 2009;329(2):410–21. 10.1016/j.ydbio.2009.02.029 19268450 PMC2677136

[6] Saudemont A Haillot E Mekpoh F: Ancestral regulatory circuits governing ectoderm patterning downstream of Nodal and BMP2/4 revealed by gene regulatory network analysis in an echinoderm. *PLoS Genet.* 2010;6(12):e1001259. 10.1371/journal.pgen.1001259 21203442 PMC3009687

[7] Peter IS Davidson EH: The endoderm gene regulatory network in sea urchin embryos up to mid-blastula stage. *Dev Biol.* 2010;340(2):188–99. 10.1016/j.ydbio.2009.10.037 19895806 PMC3981691

[8] Li E Materna SC Davidson EH: New regulatory circuit controlling spatial and temporal gene expression in the sea urchin embryo oral ectoderm GRN. *Dev Biol.* 2013;382(1):268–79. 10.1016/j.ydbio.2013.07.027 23933172 PMC3783610

[9] Li E Cui M Peter IS: Encoding regulatory state boundaries in the pregastrular oral ectoderm of the sea urchin embryo. *Proc Natl Acad Sci U S A.* 2014;111(10):E906–13. 10.1073/pnas.1323105111 24556994 PMC3956148

[10] Davidson EH Levine MS: Properties of developmental gene regulatory networks. *Proc Natl Acad Sci U S A.* 2008;105(51):20063–6. 10.1073/pnas.0806007105 19104053 PMC2629280

[11] Davidson EH: Network design principles from the sea urchin embryo. *Curr Opin Genet Dev.* 2009;19(6):535–40. 10.1016/j.gde.2009.10.007 19913405 PMC3428372

[12] Peter IS Davidson EH: Genomic Control Process. Development and Evolution. Academic Press, San Diego,2015 Reference Source

[13] Oliveri P Davidson EH McClay DR: Activation of *pmar1* controls specification of micromeres in the sea urchin embryo. *Dev Biol.* 2003;258(1):32–43. 10.1016/S0012-1606(03)00108-8 12781680

[14] Oliveri P Walton KD Davidson EH: Repression of mesodermal fate by *foxa*, a key endoderm regulator of the sea urchin embryo. *Development.* 2006;133(21):4173–81. 10.1242/dev.02577 17038513

[15] Duboc V Röttinger E Besnardeau L: Nodal and BMP2/4 signaling organizes the oral-aboral axis of the sea urchin embryo. *Dev Cell.* 2004;6(3):397–410. 10.1016/S1534-5807(04)00056-5 15030762

[16] Luo YJ Su YH: Opposing nodal and BMP signals regulate left-right asymmetry in the sea urchin larva. *PLoS Biol.* 2012;10(10):e1001402. 10.1371/journal.pbio.1001402 23055827 PMC3467216

[17] Lyons DC Martik ML Saunders LR: Specification to biomineralization: following a single cell type as it constructs a skeleton. *Integr Comp Biol.* 2014;54(4):723–33. 10.1093/icb/icu087 25009306 PMC4229890

[18] Martik ML McClay DR: Deployment of a retinal determination gene network drives directed cell migration in the sea urchin embryo. *eLife.* 2015;4: pii: e08827. 10.7554/eLife.08827 26402456 PMC4621380

[19] Howard-Ashby M Materna SC Brown CT: Gene families encoding transcription factors expressed in early development of *Strongylocentrotus purpuratus*. *Dev Biol.* 2006;300(1):90–107. 10.1016/j.ydbio.2006.08.033 17054934

[20] Howard-Ashby M Materna SC Brown CT: Identification and characterization of homeobox transcription factor genes in *Strongylocentrotus purpuratus*, and their expression in embryonic development. *Dev Biol.* 2006;300(1):74–89. 10.1016/j.ydbio.2006.08.039 17055477

[21] Materna SC Howard-Ashby M Gray RF: The C _2_H _2_ zinc finger genes of *Strongylocentrotus purpuratus* and their expression in embryonic development. *Dev Biol.* 2006;300(1):108–20. 10.1016/j.ydbio.2006.08.032 16997293

[22] Bradham CA Foltz KR Beane WS: The sea urchin kinome: a first look. *Dev Biol.* 2006;300(1):180–93. 10.1016/j.ydbio.2006.08.074 17027740

[23] Lapraz F Röttinger E Duboc V: RTK and TGF-beta signaling pathways genes in the sea urchin genome. *Dev Biol.* 2006;300(1):132–52. 10.1016/j.ydbio.2006.08.048 17084834 PMC12337106

[24] Croce JC Wu SY Byrum C: A genome-wide survey of the evolutionarily conserved Wnt pathways in the sea urchin *Strongylocentrotus purpuratus*. *Dev Biol.* 2006;300(1):121–31. 10.1016/j.ydbio.2006.08.045 17069790 PMC1780136

[25] Walton KD Croce JC Glenn TD: Genomics and expression profiles of the Hedgehog and Notch signaling pathways in sea urchin development. *Dev Biol.* 2006;300(1):153–64. 10.1016/j.ydbio.2006.08.064 17067570 PMC1880897

[26] Bolouri H Davidson EH: Modeling DNA sequence-based *cis*-regulatory gene networks. *Dev Biol.* 2002;246(1):2–13. 10.1006/dbio.2002.0617 12027430

[27] Longabaugh WJ Davidson EH Bolouri H: Visualization, documentation, analysis, and communication of large-scale gene regulatory networks. *Biochim Biophys Acta.* 2009;1789(4):363–74. 10.1016/j.bbagrm.2008.07.014 18757046 PMC2762351

[28] Andrikou C Pai CY Su YH: Logics and properties of a genetic regulatory program that drives embryonic muscle development in an echinoderm. *eLife.* 2015;4:e07343. 10.7554/eLife.07343 26218224 PMC4549668

[29] Barsi JC Li E Davidson EH: Geometric control of ciliated band regulatory states in the sea urchin embryo. *Development.* 2015;142(5):953–61. 10.1242/dev.117986 25655703 PMC4352983

[30] Lapraz F Haillot E Lepage T: A deuterostome origin of the Spemann organiser suggested by Nodal and ADMPs functions in Echinoderms. *Nat Commun.* 2015;6: 8434. 10.1038/ncomms9434 26423516 PMC4600745

[31] Sherwood DR McClay DR: Identification and localization of a sea urchin Notch homologue: insights into vegetal plate regionalization and Notch receptor regulation. *Development.* 1997;124(17):3363–74. 9310331 10.1242/dev.124.17.3363

[32] Sherwood DR McClay DR: LvNotch signaling mediates secondary mesenchyme specification in the sea urchin embryo. *Development.* 1999;126(8):1703–13. 10079232 10.1242/dev.126.8.1703

[33] Sweet HC Gehring M Ettensohn CA: LvDelta is a mesoderm-inducing signal in the sea urchin embryo and can endow blastomeres with organizer-like properties. *Development.* 2002;129(8):1945–55. 11934860 10.1242/dev.129.8.1945

[34] Peter IS Davidson EH: A gene regulatory network controlling the embryonic specification of endoderm. *Nature.* 2011;474(7353):635–9. 10.1038/nature10100 21623371 PMC3976212

[35] Wikramanayake AH Huang L Klein WH: beta-Catenin is essential for patterning the maternally specified animal-vegetal axis in the sea urchin embryo. *Proc Natl Acad Sci U S A.* 1998;95(16):9343–8. 10.1073/pnas.95.16.9343 9689082 PMC21340

[36] Logan CY Miller JR Ferkowicz MJ: Nuclear beta-catenin is required to specify vegetal cell fates in the sea urchin embryo. *Development.* 1999;126(2):345–57. 9847248 10.1242/dev.126.2.345

[37] Weitzel HE Illies MR Byrum CA: Differential stability of beta-catenin along the animal-vegetal axis of the sea urchin embryo mediated by dishevelled. *Development.* 2004;131(12):2947–56. 10.1242/dev.01152 15151983

[38] Sethi AJ Angerer RC Angerer LM: Gene regulatory network interactions in sea urchin endomesoderm induction. *PLoS Biol.* 2009;7(2):e1000029. 10.1371/journal.pbio.1000029 19192949 PMC2634790

[39] Croce J Range R Wu SY: Wnt6 activates endoderm in the sea urchin gene regulatory network. *Development.* 2011;138(15):3297–306. 10.1242/dev.058792 21750039 PMC3133919

[40] Sethi AJ Wikramanayake RM Angerer RC: Sequential signaling crosstalk regulates endomesoderm segregation in sea urchin embryos. *Science.* 2012;335(6068):590–3. 10.1126/science.1212867 22301319 PMC4827163

[41] Range RC Angerer RC Angerer LM: Integration of canonical and noncanonical Wnt signaling pathways patterns the neuroectoderm along the anterior-posterior axis of sea urchin embryos. *PLoS Biol.* 2013;11(1):e1001467. 10.1371/journal.pbio.1001467 23335859 PMC3545869

[42] Duboc V Lapraz F Saudemont A: Nodal and BMP2/4 pattern the mesoderm and endoderm during development of the sea urchin embryo. *Development.* 2010;137(2):223–35. 10.1242/dev.042531 20040489

[43] Haillot E Molina MD Lapraz F: The Maternal Maverick/GDF15-like TGF-β Ligand Panda Directs Dorsal-Ventral Axis Formation by Restricting Nodal Expression in the Sea Urchin Embryo. *PLoS Biol.* 2015;13(9):e1002247. 10.1371/journal.pbio.1002247 26352141 PMC4564238

[44] Duboc V Röttinger E Lapraz F: Left-right asymmetry in the sea urchin embryo is regulated by nodal signaling on the right side. *Dev Cell.* 2005;9(1):147–58. 10.1016/j.devcel.2005.05.008 15992548

[45] Walton KD Warner J Hertzler PH: Hedgehog signaling patterns mesoderm in the sea urchin. *Dev Biol.* 2009;331(1):26–37. 10.1016/j.ydbio.2009.04.018 19393640 PMC2702090

[46] Bessodes N Haillot E Duboc V: Reciprocal signaling between the ectoderm and a mesendodermal left-right organizer directs left-right determination in the sea urchin embryo. *PLoS Genet.* 2012;8(12):e1003121. 10.1371/journal.pgen.1003121 23271979 PMC3521660

[47] Materna SC Swartz SZ Smith J: Notch and Nodal control forkhead factor expression in the specification of multipotent progenitors in sea urchin. *Development.* 2013;140(8):1796–806. 10.1242/dev.091157 23533178 PMC3621494

[48] Armstrong N Hardin J McClay DR: Cell-cell interactions regulate skeleton formation in the sea urchin embryo. *Development.* 1993;119(3):833–40. 8187642 10.1242/dev.119.3.833

[49] Duloquin L Lhomond G Gache C: Localized VEGF signaling from ectoderm to mesenchyme cells controls morphogenesis of the sea urchin embryo skeleton. *Development.* 2007;134(12):2293–302. 10.1242/dev.005108 17507391

[50] Röttinger E Saudemont A Duboc V: FGF signals guide migration of mesenchymal cells, control skeletal morphogenesis [corrected] and regulate gastrulation during sea urchin development. *Development.* 2008;135(2):353–65. 10.1242/dev.014282 18077587

[51] Adomako-Ankomah A Ettensohn CA: Growth factor-mediated mesodermal cell guidance and skeletogenesis during sea urchin gastrulation. *Development.* 2013;140(20):4214–25. 10.1242/dev.100479 24026121

[52] Duboc V Lapraz F Besnardeau L: Lefty acts as an essential modulator of Nodal activity during sea urchin oral-aboral axis formation. *Dev Biol.* 2008;320(1):49–59. 10.1016/j.ydbio.2008.04.012 18582858

[53] Lapraz F Besnardeau L Lepage T: Patterning of the dorsal-ventral axis in echinoderms: insights into the evolution of the BMP-chordin signaling network. *PLoS Biol.* 2009;7(11):e1000248. 10.1371/journal.pbio.1000248 19956794 PMC2772021

[54] Bradham CA Oikonomou C Kühn A: Chordin is required for neural but not axial development in sea urchin embryos. *Dev Biol.* 2009;328(2):221–33. 10.1016/j.ydbio.2009.01.027 19389361 PMC2700341

[55] McIntyre DC Seay NW Croce JC: Short-range Wnt5 signaling initiates specification of sea urchin posterior ectoderm. *Development.* 2013;140(24):4881–9. 10.1242/dev.095844 24227654 PMC3848187

[56] McIntyre DC Lyons DC Martik M: Branching out: origins of the sea urchin larval skeleton in development and evolution. *Genesis.* 2014;52(3):173–85. 10.1002/dvg.22756 24549853 PMC3990003

[57] Cui M Siriwon N Li E: Specific functions of the Wnt signaling system in gene regulatory networks throughout the early sea urchin embryo. *Proc Natl Acad Sci U S A.* 2014;111(47):E5029–38. 10.1073/pnas.1419141111 25385617 PMC4250154

[58] Piacentino ML Ramachandran J Bradham CA: Late Alk4/5/7 signaling is required for anterior skeletal patterning in sea urchin embryos. *Development.* 2015;142(5):943–52. 10.1242/dev.114322 25633352

[59] Ettensohn CA: Lessons from a gene regulatory network: echinoderm skeletogenesis provides insights into evolution, plasticity and morphogenesis. *Development.* 2009;136(1):11–21. 10.1242/dev.023564 19060330

[60] Ettensohn CA: Encoding anatomy: developmental gene regulatory networks and morphogenesis. *Genesis.* 2013;51(6):383–409. 10.1002/dvg.22380 23436627

[61] Saunders LR McClay DR: Sub-circuits of a gene regulatory network control a developmental epithelial-mesenchymal transition. *Development.* 2014;141(7):1503–13. 10.1242/dev.101436 24598159 PMC3957374

[62] Rafiq K Shashikant T McManus CJ: Genome-wide analysis of the skeletogenic gene regulatory network of sea urchins. *Development.* 2014;141(4):950–61. 10.1242/dev.105585 24496631

[63] McClay DR: Evolutionary crossroads in developmental biology: sea urchins. *Development.* 2011;138(13):2639–48. 10.1242/dev.048967 21652646 PMC3109595

[64] Erkenbrack EM Davidson EH: Evolutionary rewiring of gene regulatory network linkages at divergence of the echinoid subclasses. *Proc Natl Acad Sci U S A.* 2015;112(30):E4075–84. 10.1073/pnas.1509845112 26170318 PMC4522742

[65] Gao F Thompson JR Petsios E: Juvenile skeletogenesis in anciently diverged sea urchin clades. *Dev Biol.* 2015;400(1):148–58. 10.1016/j.ydbio.2015.01.017 25641694

[66] Wray GA McClay DR: The origin of spicule-forming cells in a 'primitive' sea urchin ( *Eucidaris tribuloides*) which appears to lack primary mesenchyme cells. *Development.* 1988;103(2):305–15. 3066611 10.1242/dev.103.2.305

[67] McCauley BS Wright EP Exner C: Development of an embryonic skeletogenic mesenchyme lineage in a sea cucumber reveals the trajectory of change for the evolution of novel structures in echinoderms. *Evodevo.* 2012;3(1):17. 10.1186/2041-9139-3-17 22877149 PMC3482387

[68] Hinman VF Nguyen AT Cameron RA: Developmental gene regulatory network architecture across 500 million years of echinoderm evolution. *Proc Natl Acad Sci U S A.* 2003;100(23):13356–61. 10.1073/pnas.2235868100 14595011 PMC263818

[69] Hinman VF Nguyen A Davidson EH: Caught in the evolutionary act: precise *cis*-regulatory basis of difference in the organization of gene networks of sea stars and sea urchins. *Dev Biol.* 2007;312(2):584–95. 10.1016/j.ydbio.2007.09.006 17956756

[70] Yankura KA Martik ML Jennings CK: Uncoupling of complex regulatory patterning during evolution of larval development in echinoderms. *BMC Biol.* 2010;8:143. 10.1186/1741-7007-8-143 21118544 PMC3002323

